# Supplementary material for: Attosecond-Angstrom free-electron-laser towards the cold beam limit
Source: Nat Commun. 2023 Feb 24;14:1054. doi: 10.1038/s41467-023-36592-z (PMC9958197; doi:10.1038/s41467-023-36592-z)
Supplement: Supplementary file 1 — Supplementary Information [file 41467_2023_36592_MOESM1_ESM.pdf]

Supplementary Information

Attosecond-Angstrom free-electron-laser towards the cold beam limit

A. F. Habib[1,2*], G.G. Manahan[1,2], P. Scherkl[1,2,3], T. Heinemann[1,2], A. Sutherland[1,2], R. Altuiri[1,4], B. M. Alotaibi[1,4], M. Litos[5], J. Cary[5,6], T. Raubenheimer[7], E. Hemsing[7], M. J. Hogan[7], J.B. Rosenzweig[8], P. H. Williams[2,9], B. W. J. McNeil[1,2] and B. Hidding[1,2,10*]

[1] Scottish Universities Physics Alliance, Department of Physics, University of Strathclyde, Glasgow, UK
[2] The Cockcroft Institute, Daresbury, UK
[3] University Medical Center Hamburg - Eppendorf, University of Hamburg, 20246 Hamburg, Germany
[4] Physics Department, Princess Nourah Bint Abdulrahman University, Riyadh, KSA
[5] Center for Integrated Plasma Studies, Department of Physics, University of Colorado, Boulder, Colorado, USA
[6] Tech-X Corporation, Boulder, USA
[7] SLAC National Accelerator Laboratory, Menlo Park, California, USA
[8] Department of Physics and Astronomy, University of California Los Angeles, USA
[9] ASTeC, STFC Daresbury Laboratory, Warrington, UK
[10] Institute for Laser and Plasma Physics, Heinrich-Heine-University Düsseldorf, Germany

*_Corresponding authors:_
ahmad.habib@strath.ac.uk; bernhard.hidding@uni-duesseldorf.de

Supplementary Figures

Supplementary Figure 1 shows the averaged slice witness emittance and energy spread stability as key beam quality parameters and performance indicators for the subsequent $\lambda_r \approx 1.5$ Å X-FEL process, over the entire 23 m length of the plasma-X-FEL. At the start of the 3D PIC simulation the plasma density is ramped up to its maximum value and remains constant until the extraction section (Supplementary Figure 1a). After 2.5 cm of acclimatization phase, where the driver and the plasma tune in, the witness beam is released by a plasma photocathode. After the formed witness beam is trapped within the wakefield with 100% charge capture efficiency, its slice emittance arrives and remains at the 20 nm-rad level, while the slice energy spread decreases adiabatically with increasing beam energy. At the plasma exit, an extraction plasma density down ramp releases the witness beam into vacuum with normalized averaged slice emittance $\varepsilon_{n,(x,y)} \approx 20$ nm-rad and averaged slice energy spread $\Delta W/W \approx 0.04$ % (Supplementary Figure 1b and 1c).

The subsequent transport line captures and collimates (PMQ triplet), isolates (chicane), and refocuses (EMQ-triplet) the witness beam without quality degradation. Normalized slice emittance and slice energy spread remain at the initial values after the ~10 m transport line (Supplementary Figure 1b and 1c). This nm-rad level emittance and 0.01% level slice energy spread preservation is an unprecedented feature of the present plasma-X-FEL approach. The EMQ triplet focuses the witness beam achromatically into the undulator section (Supplementary Figure 1b). The electron beam reaches its focal point at $z \approx 17.5$ m in the undulating plane at a beam size $\sigma_y \approx 3.0$ µm corresponding to $\beta^* \approx 2.4$ m, this is approximately at the exponential gain region of the radiation amplification (Supplementary Figure 1c). The additional focusing on the y-plane comes from the natural undulator focusing.

The SASE radiation gain starts with the typical lethargy regime where in the first few power gain lengths up to $z \approx 16.0$ m the X-FEL is in the start-up mode (Supplementary Figure 1c). When the FEL process kicks in, the radiation gain increases dramatically in few metres and saturates at around $z \approx 21.0$ m. Due to the energy exchange between electron beam and radiation pulse, the slice energy spread of the beam increases by a factor of two $(\Delta W/W) \approx 0.05$ % at saturation; the observed increase is approximately of the order of the FEL efficiency parameter ρ. Meanwhile, the normalized slice emittance of the electron beam stays nearly at its initial value of 20 nm-rad. Jointly, this means that the quality of the electron beam is still extremely high even after the X-FEL interaction, thanks to the initial electron beam quality budget. Witness beam parameters at relevant positions are summarized in Supplementary Table 1, and Supplementary Table 2 condenses corresponding plasma-X-FEL performances for the two cases presented in this work. It is worthwhile to emphasize that the slice emittance growth from injection to the undulator entrance is of the order of few nm-rad (see Supplementary Table 1).

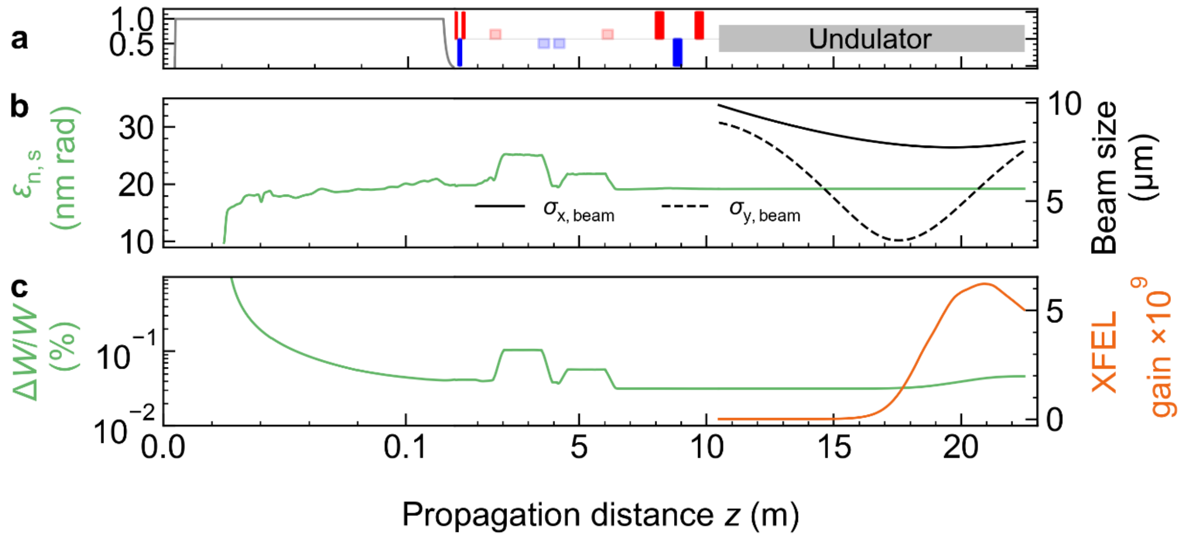

Supplementary Figure 1 | Key witness beam parameter evolution along the plasma-X-FEL. a Representation of different building blocks of the plasma-X-FEL. From left to right, the plasma density profile (grey solid line), electron beam transport line elements (red and blue blocks), and undulator section (grey block) are shown. **b** Normalized average slice emittance evolution over the 23 m propagation distance (green solid line), and witness beam focusing inside the undulator (black solid (horizontal plane) and dashed (vertical plane) line). **c** Slice energy spread evolution along the three building blocks (green solid line) and X-FEL gain curve in the undulator (orange solid line).

In conventional averaged FEL codes with slowly varying envelope approximation, only spectral modes close to the resonance frequency f_r can be resolved accurately. The Nyquist condition $f_r/2 < f < 3\, f_r/2$ provides the confidence range of frequencies in these codes[1]. The unaveraged integration of the FEL equations in Puffin enables the capability to model the radiation spectrum over an extended range simultaneously. This allows self-consistent modeling of higher harmonics. The upper frequency limit in Puffin is given by the Nyquist frequency $f_N = f_s/2$, where f_s is the sampling rate of the electro-magnetic field. It means that wavelengths down to $\lambda_N = 2\lambda_s$ can be accurately resolved, where λ_s is the discretization cell length. In the present configuration with $\lambda_s = \lambda_r/10$ (see Methods), up to the 5th harmonics λ =

λ_r/5 can be computed with confidence. Here, we set the wavelength cut-off at the 4th harmonics due to increasing computing demand for shorter wavelength at higher harmonics. Overall, this makes the modelling of the FEL process extremely accurate and provides additional insights into the physics of the FEL interaction. Supplementary Figure 2 shows the unfiltered spectra of the two respective cases modeled in this work. One can see the fundamental modes for n = 1, namely $\lambda_r = \lambda_u/(n2\gamma^2)[1+K^2/2] \approx 1.5$ Å and $\lambda_r \approx 0.8$ Å in Supplementary Figure 2a and 2b, respectively. We can also see that due to the clean X-FEL process, sideband amplification is strongly minimized near the pronounced spectral peaks and beyond. Further, excitation of higher harmonics up to cut-off n = 4 are present. The odd on-axis harmonics with n = 1,3 are more pronounced compared with the off-axis even harmonics with n = 2,4. For the $\lambda_r \approx 0.8$ Å case, radiation mode excitation down to $\lambda_r/4 \approx 0.2$ Å is evident. This indicates that there is a potential for even shorter radiation wavelengths. Future work may elaborate on this capability for X-FEL pulse production at even shorter wavelengths.

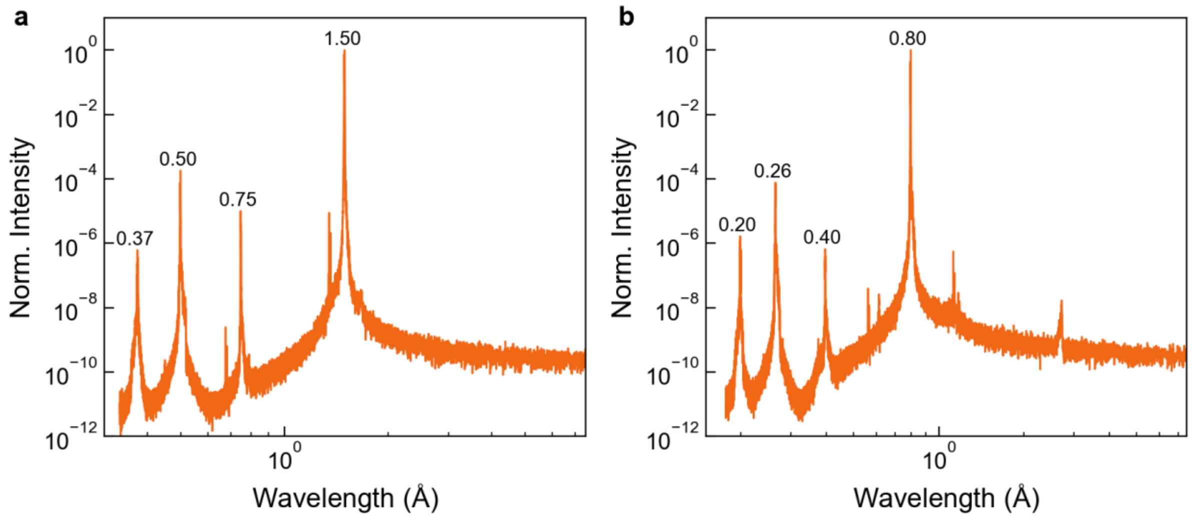

Supplementary Figure 2 | Full radiation spectra for the two X-FEL cases at saturation. The full spectra of the $\lambda_r \approx 1.5$ Å and $\lambda_r \approx 0.8$ Å cases are presented in **a** and **b**, respectively. Further even and odd harmonics following the λ_r /n scaling up to n = 4 of the fundamental modes are highlighted. The shortest radiation wavelengths observable are $\lambda_r / 4 \approx 0.37$ Å and $\lambda_r / 4 \approx 0.2$ Å for the two respective cases.

Supplementary Tables

Supplementary Table 1 | Summary of the ultra-high quality witness beam parameters at the different locations W1-W5, and incoming driver beam properties. The z-positions correspond to the scale in Supplementary Figure 1. Witness beam parameters are displayed just after plasma photocathode injection and trapping (W1), before escort bunch release (W2), at the optimum dechirping and plasma stage exit (W3), at the undulator entrance (W4) and post X-FEL interaction (W5).

	Incoming Driver	W1	W2	W3	W4	W5
Z-position (m)	0	0.032	0.08	0.12	10.5	23
Energy (GeV)	2.5	0.230	1.750	2.725	2.725	2.725
Duration (fs)	42	0.52	0.52	0.52	0.57	0.57
Peak Current (kA)	~5.5	~1.2	~1.2	~1.2	~1.2	~1.2
Projected and (slice) normalized emittance (nm-rad)	2×10^3 (2×10^3)	23 (17)	32 (~20)	45 (~20)	46.6 (~20)	46.6 (~20)
Projected and (slice) energy spread (%)	2.0 (2.0)	1.1 (0.4)	1.1 (0.05)	0.08 (0.04)	0.08 (0.026)	0.08 (0.05)
Projected and (slice) 6D brightness ($Am^{-2}rad^2$/0.1%bw)	7.0×10^{13} (7.0×10^{13})	4.0×10^{17} (1.0×10^{18})	2.0×10^{17} (6.6×10^{18})	1.3×10^{18} (7.5×10^{18})	1.3×10^{18} (1.1×10^{19})	1.3×10^{18} (6×10^{18})

Supplementary Table 2 | Summary of the plasma-X-FEL performance for the two respective cases C1 and C2 presented in this work.

	λ_u (mm)	K	λ_r (nm)	E_{ph} (keV)	ρ_{1D} $\times 10^{-4}$	L_{1D} (m)	$L_{G,th}$ (m)	$L_{G,sim}$ (m)	$P_{r,th}$ (GW)	$P_{r,sim}$ (GW)	$\Delta\tau$ (as)	β^* (m)
C1	5	1.18	0.149	8.3	7.6	0.30	0.49	0.54	4.0	4.0	~100	~2.4
C2	3	1.0	0.079	15.7	5.5	0.25	0.42	0.62	2.8	0.5	~100	~2.4

Supplementary Discussion 1: Improvements and prospects

Supplementary Figure 1 suggests that the beam quality after the FEL interaction is still extremely high. One may consider utilizing the post-FEL electron beam further, in a subsequent FEL interaction, or for other radiation production schemes, such as an ion channel laser, inverse Compton scattering, and more[2]. A multi-use exploitation would allow further overall facility efficiency and capability improvements. Non-invasive post-FEL electron beam metrology would be a desirable feature in such scenarios, and plasma afterglow-based diagnostics[3] may help provide those.

There is significant potential to further improve the witness electron beam quality demonstrated here, for example simply by working at lower plasma densities n_0. Reducing n_0 means larger blowout sizes, flatter electrostatic potential distribution and reduced wakefield amplitudes.

This has several advantages: Firstly, operating the plasma photocathode at such working points promises even better witness beam energy spreads and emittance[4]. Secondly, a larger blowout improves the relative spatiotemporal injection precision, thus increasing the stability and tunability[4,5] of witness and escort beam generation. Thirdly, the blowout regime can be already reached by driver beams with lower charge and current density, and larger matched size. This can reduce the demands put on the driver beam generation. Fourthly, when driver and wakefield electric fields are reduced, gas species with lower ionization threshold can be employed, for example hydrogen as plasma wave medium, and helium as plasma photocathode medium. This extends the tunable plasma density range of the injector and reduces the required laser intensities. However, modelling acceleration at lower plasma densities requires an even larger simulation box, and longer simulation distances to reach a target witness beam energy. This is currently unaffordable even with our simulation setup being optimized for computational efficiency and precision.

Such potential for further improvement of quality and tunability of the generated electron bunches, in turn would further extend the potential parameter range of producible photon pulses. For example, one could optimize the single-spike distinctness by decreasing the electron bunch length further, or by reducing the gain length further, or by both. Such a reduction of gain length would reduce the undulator periods required until saturation and hence reduce the slippage length. One could also improve the beam power, by increasing the electron beam energy or current. It shall be noted that while the electron beam quality in conventional X-FELs is largely optimized, novel photon pulse modalities are continually being realized, due to highly innovative ways to exploit the electron beam[6], for example via various seeding mechanisms or novel undulator configurations. Such methods are also applicable with the ultrabright electron bunches from the plasma photocathode for advanced photon pulse generation. Multi-colour X-FEL could also be realized, by producing more than one witness beam[7].

One could also attempt to push further towards harder photon energies. The diffusion rate of the energy spread scale as γ^4, and operation at reduced electron energy thus becomes crucial[8,9]. The combination of (further) reduced residual (slice) energy spread and (slice) emittance, increased brightness, and optical or advanced magnetic undulators could support generation of coherent pulses with increasingly higher photon energy. This direction is supported by the findings in Supplementary Figure 2 where higher harmonic lasing down to ≈ 0.2 Å is present within the modelled spectral range. However, towards even shorter radiation wavelength future studies need to address challenges, such as strong electromagnetic recoil effects, quantized nature of the radiation, and smaller electron density per FEL bucket. While current results are far away from the quantum FEL regime, according to the “quantum FEL parameter”[10] $\rho_{\mathrm{QFEL}} = \rho_{\mathrm{1D}}\, m_e c \gamma_r / \hbar k$ with $\rho_{\mathrm{QFEL,C1}} \approx 53.4$ and $\rho_{\mathrm{QFEL,C2}} \approx 24.1$ even for the 4th harmonics, further push towards harder photons may enter the $\rho_{\mathrm{QFEL}} < 1$ regime and will require quantum treatment of the FEL process.

The miniaturization of hard plasma-based X-FELs will not only provide additional capacities, but in combination with new capabilities will enable to employ hard X-FELs ubiquitously, for example as diagnostics for plasma, nuclear, or high energy physics experiments and novel applications.

Supplementary Discussion 2: Practical considerations

The setup length from plasma photocathode PWFA stage to the end of undulator in the demonstrated showcases amounts to only ~25 m. In case of linac-driven PWFA, the total system length would then be dominated by the linac length. However, the fact that the quality

requirements for a driver beam for a plasma photocathode PWFA system are dramatically lower in terms of emittance and energy spread as for driving an FEL directly[5], opens additional pathways for mixed mode installations. For example, linac-generated electron beams that have powered an undulator in a traditional FEL setup can still be of high enough quality to power a plasma photocathode PWFA arm as brightness transformer. Such an afterburner plasma-X-FEL extension would increase the overall capabilities and efficiency of the facility, including existing FELs.

In case of an LWFA-driven PWFA, the experimental section exploiting the X-ray pulses after the undulator may become the part with the largest spatial footprint.

The ultralow emittance of the ultrashort bunches allows operation at low electron energy, due to the emittance criterion $\varepsilon_n < \lambda_r \gamma / 4\pi$. In order to fully exploit this, ideally short-period undulators are to be used. This allows operation at hard resonance wavelengths $\lambda_r = \lambda_u/(2\gamma^2)[1+K^2/2]$ already at the low energies, yielding direct advantages e.g. for gain, saturation and cooperation length minimization, and consequentially for photon pulse generation as discussed. In contrast, beams with larger emittances cannot be used to lase at these hard resonance wavelengths in such short-period undulators; this is a regime uniquely open to ultralow emittance beams. For full performance and capability maximization, low electron energy, low emittance and short period undulators belong together. Therefore, the undulator period lengths and fields considered here are ambitious but legitimated by increasingly successful R&D in this area[11,12] and are following the trend and desire of operating the X-FEL at increasingly lower emittances, lower energy and shorter undulator periods. Longer undulator period lengths would require higher electron energies than ~2.7 GeV from PWFA for hard X-FEL realization. Should short-period undulator technology not develop sufficiently fast, the PWFA provides sufficient budget for operation at higher energies, which is an experimental reality achieved e.g. at SLAC FACET, and in turn allows operation at relaxed undulator parameters. From the simulation point of view, lower electron energies are also very helpful to facilitate full start-to-end simulation. Higher energy beams require longer particle-in-cell simulation and would have exceeded computational resources here.

Supplementary Discussion 3: Stability of electron beams from plasma photocathodes

By virtue of principle of injection around the electrostatic maximum in the centre of the plasma wave, plasma photocathodes are robust regarding spatiotemporal injection precision, which nurtures prospects for shot-to-shot stability even when the synchronization between linac-generated electron beams and plasma photocathode laser pulses amounts to tens of fs[5]. As commented in Supplementary Discussion 1, a larger blowout size improves relative spatiotemporal injection precision further. An absolute temporal injection precision improvement is provided by the intrinsic temporal synchronization in the hybrid LWFA-driven PWFA approach.

The low energy spread already achieved in-plasma not only facilitates beam transport without significant brightness loss, but also makes extraction and beam capture robust versus against potential shot-to-shot variations of the plasma downramp profile that may be encountered in experiments. This further nurtures hopes for experimentally robust quality preservation during transport of the beam.

Supplementary Discussion 4: Repetition rate and efficiency considerations

Both PWFA[13,14] as well as LWFA[15] may be operated at kHz or even MHz repetition rates; it may therefore be possible to realize the presented plasma-based X-FEL also at high repetition rates. However, more important than quantity considerations is the quality of producible output

photon pulses. For example, the number of "useful" photons is a key characteristic to consider. Here we show a clean photon pulse resulting from a well-defined electron beam with ultra-high quality. The combination of increased repetition rate in conjunction with elevated number of "useful" photons may dramatically improve the quality of experiments conducted compared with state-of-the-art X-FELs. Similarly, while wall-plug energy efficiency is important for an overall facility design, here we concentrate on the capabilities enabled by the approach. Without the brightness-transforming component of the approach, plasma-accelerated electron beams would not reach the required quality threshold for the hard X-FEL regime, and the overall efficiency in terms of coherent hard X-ray photons would be zero.

Supplementary References

[1] Campbell, L.T., McNeil, B.W.J. & Reiche, S. Two-colour free electron laser with wide frequency separation using a single monoenergetic electron beam. *New J. Phys.* **16**, 103019, (2014)

[2] Habib, A. F. et al. Plasma accelerator-based ultrabright x-ray beams from ultrabright electron beams. *Proc. of SPIE* Vol. **11110** (2019).

[3] Scherkl, P. et al. Plasma photonic spatiotemporal synchronization of relativistic electron and laser beams. *Phys. Rev. Accel. Beams* **25**, 052803 (2022)

[4] Manahan, G. & Habib, A.F. et al. Single-stage plasma-based correlated energy spread compensation for ultrahigh 6D brightness electron beams. *Nat. Comm.* **8**, 15705 (2017).

[5] Habib, F. A. et al. Ultrahigh brightness beams from plasma photoguns. Preprint at *https://arxiv.org/abs/2111.01502* (2021).

[6] McNeil, B.W.J., & Thompson, N.R. X-ray free-electron lasers. *Nat. Phot.* **4**, 814 (2010).

[7] Hidding, B. et al. Tunable Electron Multibunch Production in Plasma Wakefield Accelerators. *arXiv* 1403.1109 (2014).

[8] Huang, Z & Kim, K.-J. Review of x-ray free-electron laser theory. *Phys. Rev. ST Accel. Beams* **10**, 034801 (2007).

[9] Saldin, E. L., Schneidmiller, E. A., & Yurkov, M. V. Calculation of energy diffusion in an electron beam due to quantum fluctuations of undulator radiation. *Nuclear Instruments and Methods in Physics Research Section A: Accelerators, Spectrometers, Detectors and Associated Equipment*, **381(2-3)**, 545-547 (1996).

[10] Bonifacio, R. et. al. Quantum regime of free electron lasers starting from noise. *Phys. Rev. ST Accel. Beams* **9**, 090701 (2006).

[11] Nguyen, F. et al. XLS deliverable D5. 1–Technologies for the compactlight undulator. *XLS-Report-2019-004* (2019).

[12] Rosenzweig, J.B. et al. An ultra-compact x-ray free-electron laser. *New J. Phys.* **22**, 093067 (2020).

[13] Gilljohann, M. F. et al. Direct observation of plasma waves and dynamics induced by laser-accelerated electron beams. *Physical Review* **X 9.1**, 011046 (2019).

[14] D'Arcy, R. et al. Recovery time of a plasma-wakefield accelerator. *Nature* **603**, 58–62 (2022).

[15] Rovige, L. et al. Demonstration of stable long-term operation of a kilohertz laser-plasma accelerator. *Phys. Rev. Accel. Beams.* **23**, 093401 (2020).
